# The black hole of dealing with a disability diagnosis: Views of South African rural parents

**DOI:** 10.4102/ajod.v10i0.951

**Published:** 2021-11-29

**Authors:** Vuyelwa V. Duma, Ntombekhaya Tshabalala, Gubela Mji

**Affiliations:** 1 Happy home Children Centre, Umtata, South Africa; 2 Centre for Disability and Rehabilitation Studies, Department of Global Health, Faculty of Health Sciences, Stellenbosch University, Cape Town, South Africa; 3 Imijeloyophuhliso Foundation, East London, South Africa

**Keywords:** children with disability, parents, rural, support, South Africa

## Abstract

**Background:**

Lack of support systems in the management of health and rehabilitation related problems, including the stigma of giving birth to a child with disability, results in some parents ignoring the doctor’s prognosis of lifelong disability.

**Objectives:**

The study was conducted in the Eastern Cape province (ECP) of South Africa (SA) on parents’ views in caring for children with disability in an area with minimal health facilities in a rural setting.

**Method:**

Data was collected using exploratory descriptive qualitative methods. A Xhosa-speaking researcher facilitated six focus group discussions and conducted one individual in-depth interview with 37 parents or caregivers of children with disability residing at Happy home. Only one father was interviewed. Thematic analysis was used in interpreting data obtained from interviews.

**Results:**

The findings revealed themes indicating key concerns of parents, which were as follows: challenges with disability diagnosis, negative attitudes of health professionals, health and rehabilitation related problems, and lack of support from families and community.

**Conclusion:**

Caring for children with disability in a rural setting where services are minimal or not available to the poorest people who mostly need such services is not easy. Thus, to respond appropriately to the health and support needs of children with disability, it is crucial to understand the social context and needs of their families and caregivers. Due to size of the study, findings cannot be generalised. Recommendations are made for further studies to explore the vital issues affecting parents of children with disabilities.

## Introduction and background

The diagnosis of disability in a child presents challenges for many parents and families (Huang, Kellett & St John 2010; Tigere & Makhubele 2019; Yaacob et al. 2021). For most parents on learning about their children’s disability, shock becomes the first response to dealing with the diagnosis (Hemming & Akhurst 2009; Yaacob et al. 2021), followed by refusal to accept the diagnosis, anger and fear of the unknown world that still needs to be travelled (Huang et al. 2010). Davis suggests that those who care for people with disabilities are expected to mourn and go through some form of grief on hearing the disability diagnosis. This is because disability is viewed by the rest of the society as a tragedy (Davis 1987).

Kubler-Ross and Kessler (2004) note five stages of grief that can be transferable depending on the circumstances that one is going through. These are denial, anger, bargaining, depression, and acceptance. Hemming and Akhurst (2009) assert that the way professionals disclose a child’s condition and the period taken to support the parents in dealing with the challenges that come with having a child with a disability, often puts parents in a dilemma. They further refer to this time as a ‘black hole’ as parents of children with disability grapple with the diagnosis and sometimes find themselves denying what they see (Hemming & Akhurst 2009). For these parents, denial and fear of being stigmatised is about protecting their children from social marginalisation and from being considered less valuable than children born without disabilities (Tigerera & Makhubele 2019; Tshabalala 2014; Yaacob et al. 2021). This state of denial can result in helplessness and hinders parents in planning how they will cope with the child with disability (Hemming & Akhurst 2009).

Gona et al. (2018) assert that health professionals underestimate the emotional distress and need for information experienced by parents and carers of children with disability. This emotional distress is further amplified by social factors such as fear for the future, stress, rumour-mongering and poverty (Gona et al. 2011). This is often the case for poor families living in areas that hold strongly to traditional customs and religion (Tigerera & Makhubele 2019; Tshabalala 2014). Different cultural perceptions and societal understanding of health and disability might mean that the access and provision of health and social services for children with disability by their parents and family members might vary (Legg & Penn 2013; Maart & Jelsma 2014). Depending on one’s belief system, some of the responses from a parent of a child with disability might include some visitations to one or all of the following: biomedical and/or allopathic health practitioners; indigenous healers; and listening to and gaining advice on belief-systems, some of which might be traditional, such as witchcraft and religious belief systems, to search for a cure. These parents are looking for something that might help and improve their situation. (Aldersey 2012; Tigerera & Makhubele 2019; Tshabalala 2014).

Dura-Vila (2010) suggests that raising a child with disability has significant consequences for parents and family. One possible consequence that a parent may face is the frustration of being deserted by their partner once there is a clear diagnosis that the child is permanently impaired; most of the time it is the mother who is left with a child with disability (Dura-Vila 2010; Ingstad 1997). In the absence of fathers, deserted mothers must fend for themselves, and this can result in both emotional and physical strain (Zuurmond et al. 2018).

Ingstad (1997), in the study conducted in Botswana, stated that in a poor resource setting, women who were parents or caregivers of children with disabilities were more often unemployed and were single parents who depended on their extended families for coping with a child with disability. The extended families, who are usually expected to offer support during times of crisis, often distance themselves from both the mother and the child with disability. In most cases, the mother and the child become the black sheep in their families (Tshabalala 2014; Yaacob et al. 2021).

In some instances, where the mother remains integrated with the family, some family members even go to the extent of displaying negative behaviour by calling the child names that are derogatory and destructive to the child (Tshabalala 2014). Speculation about the child’s disability amongst community members could possibly result from the lack of proper information on what causes a disability (Gona et al. 2011; Taderera & Hall 2017). As previous studies in Kilifi and Limpompo have found that disability is associated with evil spirits, punishment from God or witchcraft, this could possibly explain the aspect of people spreading rumours (Gona et al. 2011; Tigere & Makhubele 2019).

Most importantly, the issue of access to healthcare services is a major challenge. (Vergunst et al. 2017; Yaacob et al. 2021). Vergunst et al. (2015) in his article entitled *You Carry Your Own Wheelchair*, highlighted the plight faced by wheelchair users in taxis in the rural areas of South Africa (SA). In rural areas, poor infrastructure makes roads inaccessible and unsuitable for use of assistive devices like wheelchairs (Vergunst et al. 2015). The scarce public transport service results in persons with disability who use wheelchairs avoiding visits to healthcare services, which are usually far from people’s homes. It is also acknowledged that distances, transport, and the availability of services generally are more problematic in many rural areas (Eide et al. 2015). This becomes a problem as the child grows and becomes heavy to carry (Grut et al. 2009). Taxis and their drivers are reluctant to provide transport services for persons with disabilities, especially those who use wheelchairs (Grut et al. 2009). Even when the person ultimately reaches the clinic or hospital, there is no guarantee that they will receive the service they require because of poor service delivery (Mji et al. 2017).

Ensor and Cooper (2004) emphasise the need for researchers and policymakers to give attention in their work on ways to minimise barriers to healthcare services, especially for the poor and other vulnerable groups. The costs of access to healthcare services, lack of information and cultural barriers may impede these groups from benefiting from public healthcare services (Taderera & Hall 2017). For mothers of disabled children, who are already facing challenges of poverty and inadequate access to healthcare and rehabilitation services, this implies that it becomes an unsurmountable task to make further efforts to try and access healthcare and rehabilitation services (Vergunst et al. 2017).

As parents often do, despite diagnosis, they still expect their children to achieve things that in the face of society would make them proud (Van Rooyen 2002). However, because of the extent of disability, some children’s milestones become delayed and for some, impossible to achieve on their own, thus leading to parents feeling let down by their children (Van Rooyen 2002).

In rural communities such as Kenya and Malawi, parents hid their children in fear of being ridiculed. Some ill-treated them because of the overwhelming cultural pressures they encountered whilst facing the unchanging conditions of their children (Gona et al. 2011; Paget et al. 2016; Taderera & Hall 2017).

To make sense of their situations, parents, based on their faith or religion, use coping skills, and attempt to accept their children by believing that they are gifts from God (Bunning et al. 2017; Masulani-Mwale et al. 2016). In situations where parents are trying to cope with a child with disability, siblings are expected to provide care and the love that parents see their children denied by families and society at large. This can put more pressure on siblings (Hemming & Akhurst 2009). Several studies on caregiver problems in rural context confirm that, in dealing with disability diagnosis and coping with challenges, some parents resort to neglecting their children (Bunning et al. 2017; Gona et al. 2011, 2018; Paget et al. 2016; Masulani-Mwale et al. 2016). Transport costs, negative attitudes among health professional, lack of accommodation at, including accessibility to health facilities are reported as serious problems and barriers for people with disabilities in rural areas (Eider et al. 2015).

An approach that has brought positive outcomes, as reported by Gona et al. (2018), is counteracting the cultural and socio-economic challenges faced by parents or caregivers of children with disabilities by empowering rural communities with information about disability. In these spaces of empowering the community, people with disabilities can, with their experience offer unique and authentic experiences as ‘firsthand experts’ and can act as facilitators in such discussions. Despite challenges that parents and families of children with disabilities often experience, many parents of such children adapt and develop resilience in the face of challenges; particularly where parents work together and support each other in developing their own ways of addressing the challenges they often face (Gerstein et al. 2009; Gona et al. 2011; Tshabalala 2014).

Thus, if we are to respond appropriately to the health and support needs of children with disabilities, it is crucial to understand the social context and interpretation of their needs by their families and caregivers (Tshabalala 2014). This understanding of the context assists in knowing and understanding the culture, available resources – both social, health and rehabilitation – and the infrastructure of that context. This is supported by Mbwilo, Smide and Aarts (2010) in a study conducted in Tanzania, suggesting that families be empowered with skills that will facilitate and enable parents to understand the needs of their children and allow communication between the parents and a child with a disability. We conclude this introduction by drawing on Gona et al. (2011) and Bunning et al. (2017) where both articles advise that when developing programmes related to children with disability, it is important to draw from challenges faced by the parents and carers, including the values they create and priorities in adaptation to the challenges they face in caring for a child with disability. This study, therefore, aims to present parents’ views about caring for children with disability in an area with minimal health facilities in a rural setting in the Eastern Cape province (ECP) of SA.

## Methodology

### The study setting

The study was conducted at Happy home, a community, rural rehabilitation-centre (this is called Happy home throughout the study) outside Mthatha in the ECP of SA. Situated within the OR Tambo District Municipality, Mthatha is the main town of the King Sabata Dalindyebo (KSD) Local Municipality. The Municipal total area is about 1700/km^2^ and has an estimated population of 96 114 (Stats SA 2011). Mthatha is the third largest town in the ECP of SA serving as an economic and social hub servicing up to eight functionally lower-ranked towns in the region and the surrounding rural settlements (ORTDM IDP 2013). It covers about 80% of what used to be marginalised homeland in the Transkei. About two-thirds of its citizens live in poverty, with 52% being formally unemployed (Harrison 2008). The number of people living in poverty is also high (64.6%), having an unemployment rate of 65.5% and literacy rate of 42.2%. The town is generally made up of professionals, non-professionals, semi-skilled workers, unskilled workers, business people and unemployed people (Chireshe et al. 2010).

The main economic sectors include community services (55%), trade (18.5%), finance (16.9%), agriculture (3.5%), transport (3.1%), manufacturing (2.8%), and construction (2.7%) (Chireshe et al. 2010). Transport services are largely provided by the private sector with most households in KSD fully reliant on public transport.

### Study design

This is an exploratory descriptive study that utilised qualitative methods of data collection. A Xhosa-speaking researcher facilitated six focus group discussions (FGD) and conducted one individual in-depth interview with 37 parents or caregivers of children with disability residing at Happy home. Only one father was interviewed.

### Sampling

A comprehensive method of sampling was used whereby all the parents of children with disability who attended Happy home were chosen to participate in the study. The focus was to explore parents’ views on the challenges of caring for a child with disability in a rural setting. The inclusion criterion was having a child with a disability that attended Happy home. Thirty-seven parents or caregivers participated in six focus-group discussions that comprised six parents or caregivers per group. Only one father was included.

Because of the high unemployment rate, lack of resources and poor living conditions in the villages where participants reside, participants had their children with disabilities residing at Happy home during most of the year for proper care. Arrangements were made so that the Xhosa-speaking participants could travel from the surrounding villages for the FGD that were conducted at Happy Home. Feedback sessions on information collected and analysed were scheduled prior to the parents coming to collect their children for the June or July holidays. These sessions were then conducted at the same venue and the aim of the feedback workshops was to obtain confirmation from the parents about the accuracy of data which were collected earlier.

### Data collection

When parents visited Happy home for a parent meeting, they were then informed of the proposed study and invited to participate. All voluntarily agreed to participate and they, together with the researcher, worked on a schedule for interviews and meetings. Before data collection, the participants consented by signing the written consent form after the researcher had read the consent to the participants, those who could not write initialled with an x on the consent form. They were also asked to consent for FGDs and for the interview to be recorded, whilst their anonymity was also guaranteed.

The guide was used to facilitate discussions. The development of the guide was influenced by the need to respond to the aims and objectives of this study, (see last sentence in introduction). The guide facilitated a discussion from parents and covered the following questions: When did they know that their child had disability; who informed them that the child has a disability; what was their reaction; what was the response of the immediate family; and, what is the main challenge that they are experiencing. Happy home has a small hall for functions and meetings for parents or caregivers of children with disability. This is a private space that was used for conducting the FGDs. In this study, FGDs were conducted as a technique to facilitate information-sharing amongst parents.

Qualitative methods, such as FGDs and in-depth interviews, provide a ‘deeper’ understanding of social phenomena that could not be obtained through mainly quantitative methods, such as questionnaires (Morgan & Ziglio 2007; Silverman 2013). Because of the fact that the male participant was the only participant amongst female participants, an in-depth interview was conducted to ensure that he could have a private space where he was able to openly express himself. Culturally male people hardly speak in spaces that are dominated by females, and that was the case with the FGDs. It was also important to hear his views about his experience of caring for a child with disability in an area with minimal health facilities in a rural setting.

There were six FGDs that consisted mainly of mothers with children with disability and one father. The FGDs started and ended with a prayer as was the tradition of holding meetings in this area. Each FGD lasted approximately 2 h as the parents had a lot to share. The parents were able to share stories about their experiences of having to raise children with disability in rural settings and, at the same time, express their views and opinions on how they interpreted their situation. Where parents were diverting from the research questions, prompts were used to draw them back to the main aim of the study. Suitable prompts that were aligned with the questions in the guide were used to ensure that the participants remained focused on the aims and objectives of the study. The researcher opted to do the in-depth interview with the male participant as she wanted to be careful about generalising male opinions regarding raising a child with disability.

### Ethical considerations

Ethical clearance for the study was obtained from the Ethics Research Committee at Stellenbosch University, reference number: No9/06/167.

All participants completed informed consent forms, and the completed documents were received from them. The consent form explained what the study was about, including the aspects of confidentiality and anonymity. The participants were told that they were free to leave at any point without any repercussions. Furthermore, the participants were asked for their consent to audio-record the interviews.

### Reflexivity

The researcher herself is a parent of a child with disability. To avoid further biases in the study the research assistant was appointed to do transcription and translation to both languages – Xhosa and English. The research assistant was trained on key methodological aspects of the study. A priest was also appointed as research assistant to address the ethical issues of confidentiality.

## Data analysis

Data from the FGD was transcribed and translated into English as the FGD were conducted in isiXhosa as mentioned earlier. The transcription was checked by the researcher to ensure that the information on the transcripts was the same as that on the audio-CD. Each transcript from each FGD was given a number (1–6) according to the sequence of occurrence of each discussion. The transcript of the male participant was done separately and given the number 7. The data were analysed manually by the researcher.

Thematic Data Analysis became the most appropriate analysis for this study because of what Braun and Clarke (2006) describe as the advantage of its flexibility and its usefulness to provide rich, detailed yet complex accounts of data. The following six guiding steps for thematic data analysis were followed by the researcher: familiarising yourself with your data, generating initial coding, searching for themes, reviewing themes, defining and naming themes. The themes were further analysed to see how some may fit together to make up a specific theme and subthemes (Braun & Clarke 2006). On reviewing all themes and subthemes at this stage, it was important to make sure that all selected extracts were answering the research question.

The four themes which are presented in the discussion below are as follows: Challenges with disability diagnosis, Negative attitudes of the health professionals, Health- and rehabilitation-related challenges, and Lack of support from family and community. These themes and subthemes were used to present the findings.

## Presentation of findings

The themes and subthemes that emerged during the six FGDs and the single in-depth interview, were presented with direct quotations from the parents or caregivers’ expressions of their own experiences. These are presented in four main headings below. Each quotation that came from parents (P) and caregiver (C) was identified by the number assigned to that focus group discussion (FGD). Challenges with disability diagnosis; Negative attitudes of the health professionals; Health- and rehabilitation-related challenges; Lack of support from family and community. In Table 1, themes, subthemes and verbatim quotes that emerged from the participants are listed.

**TABLE 1:** Presentation of four main themes with subthemes.

Themes	Subthemes
Challenges with disability diagnosis	False blame, held accountable, culture and religion, ignoring the obvious, shifting responsibility, abandonment because of disability and health.
Negative attitudes of the health professionals	The nurses scolded me, discouraging unsupportive feedback, late diagnosis undermined future prospects.
Health- and rehabilitation-related challenges	Long distances and inaccessible healthcare services, resort not to access healthcare services, risking one’s life to gain access to health services.
Lack of support from family and community	My child was ridiculed, spiteful family members, disability myths leading to exclusion, humiliation at school led to further exclusion, resorting to fend for oneself.

Source: Adapted from Braun, V., Clarke, V., Hayfield, N. & Terry, G., 2019, *Thematic analysis,* Springer, Singapore.

### Theme 1: Challenges with disability diagnosis

The reactions of parents to disability diagnosis of a child varied in general. The responses of mothers and fathers to the birth of a child with disabilities were often negative and ultimately led to abandonment of the child by one or both parents. Below are subthemes presenting some of the challenges experienced by parents once they hear the disability diagnosis of the child.

#### Sub-theme1: False blame

The responses of mothers and fathers to the birth of a child with disability were often negative and resulted in one or both parents abandoning the child. One young mother told that she was still a student when she fell pregnant. She was not even aware that she was pregnant until the next-door neighbour brought it to the attention of her parents. She then gave birth to a child with disability and dropped out of school. The boyfriend, who was still a student at the time, rejected her after the birth of the child. She said about him:

‘Whoo! That one did not buy even the vest for the child – he said in his family there has never been a disabled person.’ (PFGD3, male teacher)

This appears to be an excuse coming from the father to justify his behaviour, thus enabling him to cope with his decision to abandon the child. The implication of such decision by a parent becomes detrimental to the child who ends up living a life of rejection. Other parents resorted to taking the child to the community-based rehabilitation centres and thereafter the child is never visited and abandoned (thus shifting the responsibility). For the children who are abandoned at Happy home, when contacted by the Centre to respond to the needs of the child, they tend to distance themselves from the child.

Other parents, especially male parents, tried to find an excuse for their behaviour of distancing themselves from the disabled child as this quote from a mother of a disabled child highlighted:

‘He said in his family there has never been a disabled person.’ (PFGD1, male teacher)

To a mother of a child with disability, this is a painful statement as within an African culture a child is seen as the extension of the family. This delinking of the child from the family by the father would be seen by the mother as a rejection of the child.

#### Sub-theme 2: Held accountable

Reintegration of the child and mother to the family after a disability diagnosis has been established is usually a problem. It appears that the family too goes through their own process of dealing with the final diagnosis. The mothers of children with disability felt not welcomed by their family members once the diagnosis was final. This rejection is more painful if it comes from the father of the child with disability as this quote from another mother of a child with disability expands:

‘He said I gave birth to a disabled child I should bear the consequences; he would never give her any support because I must suffer the consequences of having a child with disability.’ (PFGD 2, male teacher)

From the above quote, it appears that the disappointment of women giving birth to a child with disability. Fathers appear to be shifting the blame to the mothers of children with disabilities by holding back support.

#### Sub-theme 3: Culture and religion

Culture and religion played an important part in the lives of some of the participants, enabling them to accept their situations and to actively seek assistance for their children. The below extract expresses the words of a mother who faced extreme distress when she came to know that her child is disabled. But her worry was alleviated by her faith.

‘The day I was told that my child is disabled; Whoo! I was so disturbed; I did not know whether there was a missing limb because the doctor and nurse did not explain what they mean when they say the child is not right; but again, I consoled myself all creations come from God, if I do not accept that this is my child who then will?’ (PFGD4, unemployed female)

Sometimes cultural norms and religion create undue stress for mothers of children with disabilities, as so many expectations are put on mothers having children with disability. There is a general fear of the reaction of the community with all its structure and how it will respond to a child with disability. As many of the villages in rural areas are still patriarchal, it becomes better for the mother if the child is accepted by the father. Another mother had a different welcoming response from the father of the child, when asked about his reaction after the delivery of the child with disability. She said the following:

‘No Mam; there was not any bad reaction from him; he was so supportive we go to the doctor together with him and the child, even at this moment.’ (PFGD5, unemployed female)

The care and love shown by the father towards his son with disability supported the mother. That helped her to cope better with the condition of her child as she mentions the support she gets on taking the child to the doctor.

#### Sub-theme 4: Ignoring the obvious

Some of the parents refused to accept that there is some form of disability in their children.

Some of the participants told about various coping mechanisms which they and their family members used to deal with the disability diagnosis whereby they sometimes shifted the blame to witchcraft. This comment shown below from one of the family members supports this:

‘It can either be witchcraft, partners’ fault or someone else must take the blame and then take care of the child.’ (CFGD6)

The above statement showed some of the frustration of the carers and mothers of children with disability as they come back to family members seeking support. It appears that disability is not seen as a life event that happens randomly – somebody must take the blame. From this caregiver’s statement, it appears that this running around to accept the final diagnosis is linked to the need to shift the responsibility of taking care of the child with disability as it appeared that there is a need for somebody else to take the blame and that person must take care of the child.

#### Sub-theme 5: Shifting responsibility

Parents tend to distance themselves and shift the responsibilities to those taking care of the child. For example, when one parent who had her child staying at Happy home Centre for Children with disability, was contacted by the Centre to respond to the needs of the child as the child was sick, she refused to come and see the child. She said the following:

‘One day I received a phone call that I should come to Happy home to fetch him because he was sick, I was so annoyed, I asked what kind of a hostel is this?’ (PFGD1, unemployed female)

It appeared from the mother of a child with disability that the institution that was looking after her child needed to also keep the child even when the child was sick. Many of the parents stayed in rural areas where access to healthcare services was a problem; hence it appeared that this parent expected the Centre to also look after the child when the child was sick.

#### Sub-theme 6: Abandonment because of disability and health

Other instances of abandonment of children with disabilities by parents is sometimes because of health status – such as HIV or AIDS of one or both parents and the fear that the child with disability will also have a positive HIV or AIDS status – and this is compounded by blaming attitudes of who infected whom. Ultimately, this may result in the abandonment of the child. This is highlighted by the words of a grandmother below, who was left caring for the child:

‘My grandson came with my son in 2003 from Gauteng; he was one-year and one-month old. He told me that he was in love with a lady who accused him of infecting her with the disease. She dropped the child on the bed and off she went, leaving my sick son with the child.’ (CFGD3)

### Theme 2: Negative attitudes of the health professionals

The lack of support by family members and the rejection of children in communities did not deter parents from looking for alternative support in caring for their disabled children. Health professionals were generally considered to be the best source of support and information when it came to understanding issues relating to disability. As a result, parents expected doctors and nurses to offer good advice when discovering that a child has a disability. However, parents were often disappointed by the response and the kind of support received from health professionals. The participants shared their experiences as outlined in the following subsection.

#### Sub-theme 1: The nurses scolded me

One participant shared the experience of trauma that she went through when she was delivering her child in the care of health professionals. Despite telling the nurses that the baby was ready to be born, and that her waters had broken, they refused to listen to her saying the child was still far from being ready to be born. The statement below highlights some of the difficulties experienced by mothers when giving birth to their children within the public healthcare system:

‘The nurses scolded me asking if I once had a baby; didn’t I say this is my first child; they made mockery of me.’ (PFGD 2, male teacher)

These challenges sometimes led to negative consequences as this parent seem to link the disability of her child to the slow response of nurses to her need for support during delivery.

#### Sub-theme 2: Discouraging unsupportive feedback

The way feedback from health professionals is given to the parents of the child with disability regarding the disability status of the child, especially the initial diagnosis and feedback on disability status, is very important. The manner in which this is done could either give hope or dash away the parents’ hopes for the recovery or development of the child with disability. This parent attested to this:

‘What I will never forget is what the doctor told me. He said my child will never be anything; will never do anything. He crushed all hopes…. I cried a lot until my husband said the doctor is not God. The child was born normal; she may change and be something else. I don’t want to see that doctor in my life.’ (PFGD3, male teacher)

Parents were left hopeless and those with partners were assisted by their partners in dealing with the prognosis which resulted in some of the parents never wanting to see the health practitioner again. This can further undermine any future relationship with the health professional.

#### Subtheme 3: Late diagnosis undermines future prospects

A majority of the parents received the diagnosis that their child has disability quite late when the child had already started school. This would commonly come from teachers who cannot see progress in the schooling of the child. What seems to be frustrating to the parents is a lack of knowledge from the teachers for the next step the parent should follow once the diagnosis has been made. This was shared by this mother of a child with disability:

‘I was called to visit the school where I was informed that my son cannot read or write at the age of 13 years and a referral letter was written for me for the social worker to apply for a child support grant and not for disability grant who will assist her with child social support grant. At that time, the child was old enough to go to a special school, the teacher refused to write a referral letter for the child to be admitted in a special school.’ (PFGD6, unemployed female)

From this transcript, it appears that schoolteachers and their principals tend not to see the prospect and the need for further schooling of the child with disability once the child is seen as disabled. Instead, they seem to be working within the medical model which suggests that the child should be referred to the social worker for a child support grant.

### Theme 3: Health and rehabilitation related challenges

Parents/caregivers considered family and professional services as crucial contact points of support with regard to addressing and coping with the challenges of having a disabled child. However, they were often disappointed in most cases because of the inaccessibility of professional support services and lack of support from family and the larger community, as their experiences indicate here below.

#### Sub-theme 1: Long distances and accessibility to health services

The scarcity of adequate healthcare services and the long distances to health facilities in poor communities is a problem for children with disabilities. Poverty and lack of psycho-social support puts mothers of children with disabilities at risk during delivery time. One participant said:

‘I delivered at home; I was using traditional medicine because I had no money to go to the clinic… no one wanted to give me company since my mother got married to another man and left me when I was too young.’ (PFGD1, unemployed female)

Whilst another asserted that:

‘The day I will never forget is the day when I was taking my child to the clinic, which was too far, and at that particular moment she was having attack of fits.’

With the challenges of lack of health and rehabilitation services, some mothers of disabled children become so overwhelmed with those problems that they become homicidal, as confirmed by the statement below:

‘I was so scared, and I wished, I could give him poison so that he can die and be relieved from this great pain and even the clinic is too far. I thought the tank tablet [she was referring to the pesticide people locally use it to commit suicide, it is quick, people die within thirty minutes] was a quick solution; but I did not do it. I thought she is my own blood; God does not allow anyone to kill, to take away human being’s soul no matter what type of a person; is not the right thing to do, I did not do it.’ (PFGD2, unemployed female)

#### Sub-theme 2: Resort not to access health services

When asked how parents perceive the accessibility of healthcare and rehabilitation services in their areas, all seemed to be pointing to the fact that the clinics are too far away and that inadequate transportation facilities is a problem. For other parents it was a matter of choosing between the school and the healthcare service that was far away. Most of them resort to not taking their children with disability to healthcare services because of distances and transport problems as attested by the father of the child with disability:

‘I decided to keep the child at home since she is still attending mainstream school in the area. I did not see any need to start physiotherapy treatment since there is no money to transport the child everyday she needs to go to Bedford Hospital which is near the town.’ (Father of Child with disability during in-depth interview)

He ended up by saying:

‘If the Health Centres were nearer we would be happy to take our child to the hospital but under the circumstances we decided to live with the condition.’

#### Sub-theme 3: Risking one’s life to gain access to health services

The lack of access to healthcare and rehabilitation services expose parents of children with disability to risking their lives and that of the child as attested by this statement:

‘My child was suffering from toothache, the clinics were far, and there was no money to pay for the transport, I had to go out first to borrow some money and the rivers were flooded after a heavy rain I was going to cross the river to Langeni Clinic, which was too far. I was prepared to take risk alone after getting the money I borrowed from neighbours I put my child at my back going to cross the river praying for our safety. I covered my head and that of my child with my dress and through God’s grace I managed to cross the river and my child was saved from the pain of tooth ache.’ (PFGD2, unemployed female)

All the parents agreed that the problem of healthcare centres that are too far away is still a problem. They felt that in the clinics there is poor care and negative attitudes towards disability; there is no supervision and nursing staff are doing as they please.

### Theme 4: Lack of support from family and community

Extended family members were a source of support for some parents, whilst in other cases the attitudes of the extended family members were either hurtful or damaging. The abandonment of a parent by the extended family and/or a partner causes the parent to weigh up the extent of her or his loyalty between the family, partner, and the child with disability. Usually, the child with disability becomes the loser. These issues were evident in parents’ articulation of their experiences of raising a child with disability.

#### Sub-theme 1: My child was ridiculed

Many family members tend to use negative coping skills such as mockery towards the child with disability. The mother of a child affirms this about her mother in-law:

‘She would mock my child when there were visitors within the home, even when everyone was laughing because there was something amusing them; she would utter discouraging words to the child saying; “Whoo! This one is laughing louder because of her disability.”’ (PFGD5, unemployed female)

It appears that as relatives struggle with accepting that their relative child has disability, they are not sure how to cope with the child with disability especially when there are visitors.

#### Sub-theme 2: Spiteful family members

It appears that there is a general lack of understanding, attitudes, and support from family members. Having a child with disability left the family members with bad feelings, which are translated to the mother and the child, especially if the disability is visible and identifiable, as supported by the statement below:

‘The whole family was disturbed when I came back with the child from hospital, the disability was visible the child had squinty eyes.’ (PFGD5, unemployed female)

#### Sub-theme 3: Disability myths leading to exclusion

As parents/caregivers narrated their different stories, it appears that there is generally a negative attitude towards, and lack of understanding about, disability within rural South African communities. One parent shared about how challenging it was for her family raising a son with disability in a community that refused to see him as a child and accept him as they did other children.

‘Mhh! even neighbours did not want him to visit their children; because he used to go out to play with other children; parents said he must not play with others because he is something else.’ (PFGD3, unemployed female)

#### Sub-theme 4: Humiliation at school lead to further exclusion

The negative attitudes of teachers and the teasing of children with disability by their peers make children with disabilities feel excluded in their schools even if there was no problem in the family. Sadly, in schools the rejection came from both teachers and peers, as indicated in the few examples shared by parents below:

‘There was no problem in the family, only at school where she was a mockery, statements like she is stupid, senseless is even seen as she walks that she is an imbecile.’ (CFGD 3)

This is supported by the next parents:

‘The challenge was at school where they made mockery of her saying she is limping.’ (CFGD 3)

Sadly, the rejection also came from teachers, whom children often look up to and call on in times of trouble at school. This parent shared how inhumanly his teacher’s son responded when her son got involved in an accident that left him intellectually and physically disabled. The teacher responded that she was happy that the son got involved in the accident since he was then not able to attend school.

‘She said; she was relieved since he was the youngest in the class he was giving her problems.’ (PFGD3, unemployed female)

Another parent said his son was mocked by other students and they used to tease him for his lack of progress saying that;

‘He was not progressing at school and they were pointing fingers at him saying he is just coming for nothing.’ (PFGD3, unemployed female)

### Sub-theme 5: Resorting to fend for oneself

The rejection by family members results in the parents of children with disability resorting to fending for themselves. The mothers of children with disability continue to look for solutions and resources alone and unsupported. Some were able to find solutions along the way, even if they had to travel distances to obtain these solutions, as attested by the mother of a child with disability:

‘At Red Cross hospital we were given health education combined with black and white parents of children with disabilities; I discovered that the problem of disability is for all races; it has nothing to do with me and since then I started coping with her condition.’ (PFGD3, unemployed female)

## Discussion

Four themes were used to guide the discussion. These were as follows: Challenges with disability diagnosis; Negative attitudes of the health professionals; Health- and rehabilitation-related challenges; Lack of support from family and community.

### Challenges with disability diagnosis

From the findings of the study, when parents start to understand that the disability diagnosis is final they start looking for someone to blame which is usually the mother of a child with disability. This then gives the father of the child with disability an excuse to abandon both the mother and the child. Similarly, Tigerera and Makhubele (2019), Yaacob et al. (2021) and Taderera and Hall (2017), in their studies found that the majority of mothers were deserted by their partners after giving birth to a child with disability. If the father of the child with disability does not abandon his family, the next challenge would be how to integrate the child with disability into the extended family. However, if the father of the child with disability is not supportive he can end up distancing himself and his family from the child with disability by suggesting that no one in his family was ever born with disability.

For the extended family to still accept the mother and the child with disability as part of their family, this is done either by ignoring or underplaying the disability diagnosis. This is similar to other rural communities such as in Sikhukhune village in SA, Kenya and Malawi, whereby parents hid their children in fear of being ridiculed (Gona et al. 2011; Paget et al. 2016; Taderera & Hall 2017). Some ill-treated them as a result of the overwhelming cultural pressures they encountered whilst facing the unchanging conditions of their children (Gona et al. 2011; Paget et al. 2016).

Homes for children with disability such as Happy home are few in the ECP and if the child with disability is admitted to such homes this gives the family some form of relief from caring responsibilities. Such homes are usually also reasonably closer to healthcare services. But if the child gets sick, the parents of children with disability are reluctant to pick up the child because of where they stay, as they struggle to gain access to healthcare services. This can easily be seen as abandonment of the child at the time when the child most needs the attention of his or her parents. During such times, such places might feel that the parents of children with disability are shifting the caring responsibility to them.

Ensor and Cooper (2004) had highlighted the need to give attention to ways to minimise barriers to healthcare services, as these barriers can further prevent poor families from benefitting from public healthcare services. Other sicknesses such as HIV and AIDS in the family further heightens the fear that over and above the disability diagnosis of the child she or he might have contracted this condition, and this might lead to one of the parents deserting the child – especially the one who feels she or he was the one infected by the other.

All participants agreed that parents react differently to the birth of a child with disability. All parents or caregivers in the study agreed that having a child with disability was a painful experience. Some parents, however, either the mother or the father or both, abandoned the child with disability. In some cases, the responsibilities for the child with disability were then taken up by extended family members who addressed the needs of the child with disability and accessed government disability grants.

The findings in this small-scale research study coincide with several studies on caregiver problems in rural context including challenges that caregivers or parents experience in dealing with disability diagnosis and coping with challenges as highlighted in the introduction section of this article (Bunning et al. 2017; Eide et al. 2015; Ensor & Cooper 2004; Hemming & Akhurst 2009; Gona et al. 2011; Masulani-Mwale et al. 2016; Paget et al. 2016).

### Negative attitudes of the health professionals

It appears too, that in this small-scale study, teachers are caught within the medical model and tend to want to associate the child with disabilities with social workers to gain a child support grant, not a disability grant as expected. The parents on the other side would like the education of the child to continue even if it is in a special school. It appears that there is also a need to upskill teachers with regard to disability and early childhood development, especially teachers who are involved in early childhood education as, together with health professionals, they too are at the coal face of caring for the growing child (Tshabalala 2014).

The public health systems in SA are overburdened and overstretched (Mji et al. 2017; Vergunst et al. 2017). This makes the health professionals run from pillar to post trying to cope with the high volume of patients. From the participants in this study it appears that some disabilities could have been prevented if the response rate from carers within the nursing profession was attentive enough and that listening to the patient and using this as a guiding light could deliver proper caring. Blitz (2011) in his book that espouses client-centred care, emphasise the following attributes in client-centred care: there is a process of listening, observation, learning, application and practice.

The nursing profession is seen as one of the caring professions. But nurses, because of being thinly spread and working in public health systems similar to the one explained above by Mji et al. (2017) and Vergunst et al. (2017), it appears they start shutting up and speaking down to their patients, and eventually this becomes the norm. This supports Eide et al’s findings about negative attitudes of health professionals towards persons with disabilities. It is during this time that skills highlighted by Blitz (2011) disappear through the window and it is also during this time that birth mistakes easily happen. The doctors in the public sector also work under the same working conditions whereby it becomes easy to quickly give a final diagnosis that leaves parents with no hope.

The medical model further endorses this type of thinking whereby doctors tend to see disability as lack of abilities. Giving feedback in a manner that is demoralising and does not give hope to parents of children with disabilities can further discourage them and undermine the urgent need to start planning on how they will support the child with disability. Gona et al. (2018) attest that health professionals underestimate the emotional distress and the need for information experienced by the carers of children with disabilities. Together with this is a combination of social factors such as fear for the future, stress, rumour-mongering and poverty. In public health institutions, doctors are still seen as people who should role-play the important attributes of the public health model. If they present a level of lack of consideration for the parents and the child with disability it is easy for the other health professionals to imitate that type of behaviour. Bradshaw (1996) cautions that true caring is usually underpinned by qualities such as democracy, reciprocity, collaboration, and role modelling. It is also easy for some of the impairments and disabilities to manifest quite late during the growth of the child. It is also common for intellectual disabilities to be uncovered quite late and it is usually the teachers who discover this challenge.

### Health and rehabilitation related challenges

Parents or caregivers said it is difficult to access healthcare facilities when their children are sick because of the long distances they have to walk to access transport. Distances and poor roads make it difficult for parents to gain access to health and rehabilitation of their children. Sometimes the parent of a child with disability may risk their life and that of the child trying to gain access to healthcare services. Vergunst et al. (2015) highlighted the plight faced by wheelchair users in taxis in the rural areas of SA. Even when the person ultimately reaches the clinic or hospital, there is no guarantee that they will receive the service they require because of poor service delivery.

Some of the parents ultimately resort not to take the child to healthcare and rehabilitation services. This has huge implication for the future prospects of the child as the child grows and, without health and rehabilitation intervention, impairment and disability may continue to undermine the movement of the child, including other development-related activities. This lack of intervention further undermines the possibility of the child being able to gain access to basic amenities such as schooling and playing with other children (Grut et al. 2009). For mothers of disabled children who are already facing the challenges of poverty and access to health and rehabilitation services, this implies that it becomes an unsurmountable task to make further efforts to explore integration of the child with other children and the inclusion into community; so the child ends up being isolated (Bunning et al. 2017; Taderera & Hall 2017; Vergunst et al. 2017; Yaacob et al. 2021).

### Lack of support from family and community

The study revealed that, whilst a parent struggles in the process of making sense of and accepting the diagnosis of a disabled child, cultural beliefs exert more pressure on the parent rather than providing help. The stories shared by mothers of disabled children show that the extended family members mocked and taunted the disabled children in the family, causing much hurt to the parents. The parents reported that even close relatives still think that a disabled child should not be seen by visitors because she or he is an embarrassment to the family. As a result, the child becomes isolated and both the cognitive and physical development of the child gets delayed.

The cultural beliefs, norms, and values of Xhosa speakers in the rural Eastern Cape are entrenched in the society and directly affect the negative behaviour towards pregnant women and disabled children (Mji 2012; Tshabalala 2014). These beliefs, norms and values increased the stress experienced by parents or caregivers. The parents shared their different stories that revealed that these beliefs still oppress females in rural SA. These ranged from the child being bewitched to cultural values meaning that you cannot leave your in-laws no matter what. Mbwilo et al. (2010) and Hemming and Akhurst (2009) further affirm that such beliefs and responses towards mothers of children with disability are still quite common in rural SA.

A study by Tigerera and Makhubele (2019) concurred with the findings of this study that parents of children with disabilities get subjected to name labelling as they are viewed to be practising witchcraft or to be paying for the sins they committed. With regard to education, whilst within the context of inclusive education policy, teachers would ideally assist in facilitating relevant support for the parent and the child, when the child coincidentally ends up in a school setting, rejection by teachers and peer mocking caused more harm to disabled children (DoSD 2015; Gargiulo 2006; Tshabalala 2014).

## Limitations and further recommendations for this study

Because of the size of this study, the findings cannot be generalised. However, the experiences shared by the parents outline the vital issues affecting parents of children with disabilities. This could provide valuable information for further studies.

## Conclusion and recommendations

In this study, the challenges faced by parents of children with disabilities have been explored. The most prominent themes that arose were challenges with disability diagnosis, the negative attitudes of health professionals, health- and rehabilitation-related problems, and lack of support from families and community. The findings of this study have pointed strongly to the ‘black hole’ in which parents find themselves trapped in the process of accepting the disability diagnosis and responding to the needs of a child with disability. The participants confirm that it is more challenging for parents in rural settings with scarce healthcare and rehabilitation services to support the child with disability. The stigmatisation of parents by their families and communities puts more pressure on them. It also appeared to the parents of children with disability that the role that could be played by family and community in supporting children with disabilities cannot be undermined. To conclude, this was a small exploratory study. Additional researches are required to further confirm the findings of the study.
